# Forearm Hereditary Multiple Exostosis: A Retrospective Case Series Study

**DOI:** 10.7759/cureus.26039

**Published:** 2022-06-17

**Authors:** Nizar Hamdi, Hatan Mortada, Zainab Al Eid, Anas M Makhdoum

**Affiliations:** 1 Departments of Surgery and Orthopedic Surgery, King Faisal Specialist Hospital & Research Centre, Riyadh, SAU; 2 Department of Plastic Surgery & Burn Unit, King Saud Medical City, Riyadh, SAU; 3 Division of Plastic Surgery, Department of Surgery, King Saud University Medical City, Riyadh, SAU; 4 Department of Orthopaedic Surgery, King Fahad Hospital Hofuf, AlAhsa, SAU

**Keywords:** saudi arabia, benign forearm tumors, clinical experience, metachondromatosis, diaphyseal aclasis, forearm, osteochondromas, multiple hereditary exostosis

## Abstract

Background

Hereditary multiple exostosis (HME) is a significantly rare genetic condition with benign chondrogenic lesions affecting long bones. Forearm involvement is relatively common, with varied treatment modalities reported. Here we describe our experience with HME. The study is the first of its kind to be conducted in the Middle East and Saudi Arabia.

Methods

A retrospective medical record-based case review was carried out on patients with forearm HME operated from 2006 to 2022 at our institution. Patient demographics, clinical presentation, management, outcome, Masada scale, and radiological outcomes were analysed.

Results

Ten patients (12 affected forearms) with HME were included. The average age of those undergoing surgery was 12.7 ± 5.13 years, and the average length of follow-up was 62.25 months. Most patients (n = 5, 50%) had Masada type 1 (Type I indicates radial head not displaced, primary exostosis from the distal region of the ulna, ulna relatively short, radius bending). Five (50%) underwent radial head resection. The majority of the patients (n = 8, 80%) had no complications or recurrence. Two patients developed recurrence; the first one developed recurrent radial bone deformity and dislocation of the radial head and the second, who underwent excision with an iliac crest bone graft application, developed osteolysis of the bone graft with recurrent deformity.

Conclusion

HME is typically managed primarily by excision of the lesion at skeletal maturity and annual check-up and radiological follow-up. If a secondary procedure is needed in future, simple excision of the dislocated radial head would be the most feasible approach. Due to the rarity of the illness and limited literature, further studies are still required to optimize the outcome in children with HME.

## Introduction

Hereditary multiple exostosis (HME) is an autosomal dominant condition caused by a mutation in the *EXT1* or *EXT2* genes [1]. Forearm deformity is one of the commonest symptoms of HME [1]. In HME patients, the prevalence of forearm deformity varies from 40% to 74% [1]. Porter et al. found that the extent of exostosis is inversely related to the length of a bone, suggesting that local effects contribute to growth retardation [1]. Since the ulnar bone contributes more to longitudinal development and has a smaller diameter, exostosis at the distal ulna creates a more severe forearm deformity than exostosis at the radius [2]. Madelung-like deformity in the wrist, radial bending (RB), and radial head dislocation are all caused by the relative shortening of the ulna tether on the radius [3,4]. In HME, treatment recommendations for forearm deformity were quite varied. Fogel et al. and Masada et al. reported good results with combined corrective surgery that included ulnar lengthening and excision. Complications are, however, frequent with lengthening [5,6]. Simple excision improved ulnar shortening and RB [7]. Akita et al. determined that excision is the most beneficial technique for good long-term outcomes [8]. According to Shin et al., simple excision improved forearm rotation but had no effect on radiological results, and further ulnar lengthening was ineffective [9]. According to Arms et al., Noonan et al., and Litzelmann et al., patients with forearm deformities have an excellent function, with the exception of poor aesthetics [10-12]. In studies published by Akitaet al., Litzelmann et al., Tang et al., and Refsland et al., satisfactory radiological results were found for ulnar lengthening with excision [8,12-14]. In spite of the fact that multiple surgical techniques are mentioned in the literature, the most appropriate surgical method depends on a variety of factors. Therefore, this article aims to describe our experience with managing HME. To the best of the authors' knowledge, this is the first study of its kind in the Middle East describing the experience of the diagnosis, management, and natural history of HME of the forearm in 10 patients.

## Materials and methods

Ethical approval

The study was approved by the Institutional Review Board and Research Ethics Committee of King Faisal Specialist Hospital and Research Center (KFSHRC) in Riyadh, Saudi Arabia (Ref. No. 222103). This investigation adhered to the ethical principles mentioned in the Declaration of Helsinki. The patients' medical records were obtained, and data were analyzed. The patients' consent was obtained for the clinical photographs. 

Study design and patient population

In this retrospective case series study, we reviewed all patients with HME of the forearm operated at KFSHRC in Riyadh, Saudi Arabia, from 2006 to 2022. Our inclusion criteria were pediatric patients who had HME in the forearm, had not been operated on before, and did not have any other causes of the forearm deformity. The exclusion criteria were patients with forearm deformities not caused by HME or operated on before in another center.

Study groups and variables

**Figure 1:**
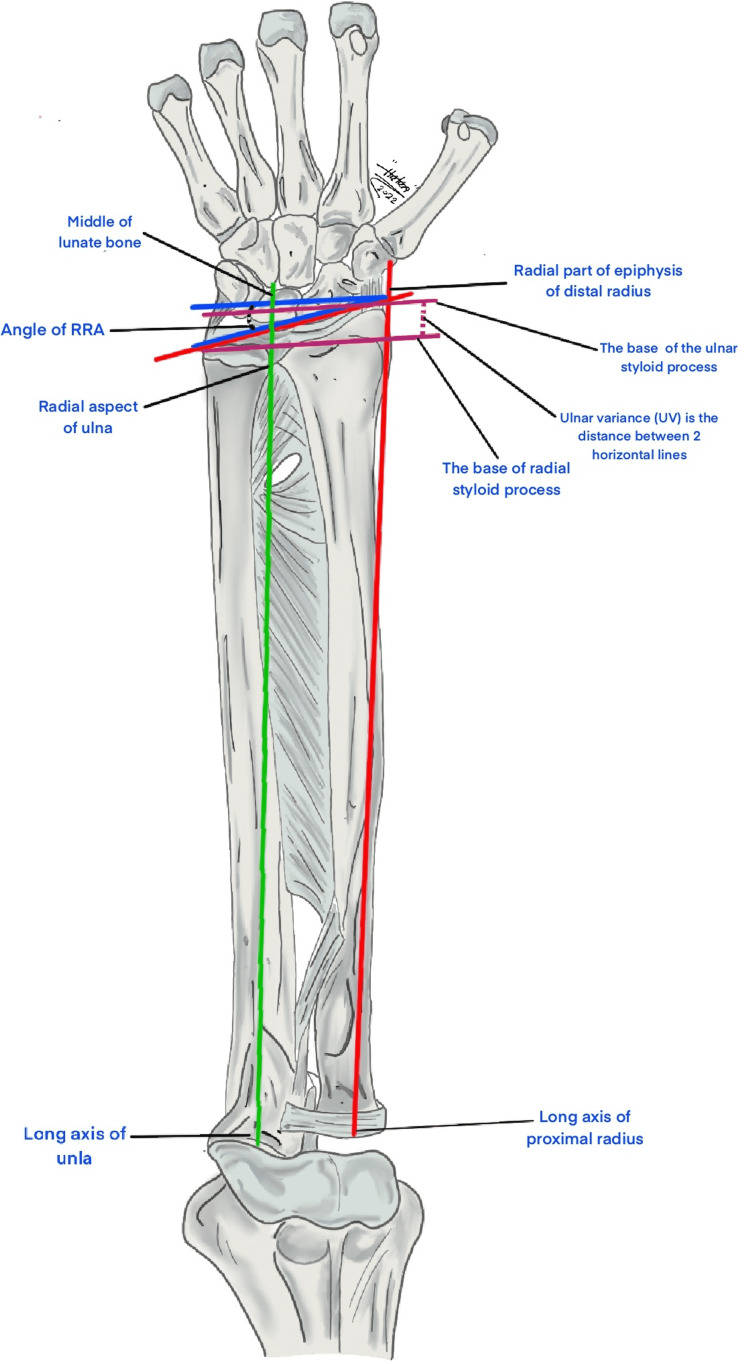
A schematic illustration shows the landmarks used to measure the different radiological data included. Ulnar variance (rose color) is the distance between two horizontal lines across the linear axes of the radius and the ulna from the internal diaphyseal plate of the radius and the base of the head of the ulna. The radioarticular angle (red color) is the angle of inclination of the distal articular surface of the radius to the long axis of the forearm. Carpal slip (green color) is the percentage of the lunate on the ulnar side of the continuation of the linear axis of the forearm. The image was illustrated by Dr. Hatan Mortada, the second author RAA: radial articular angle

Ten patients were included in our study. Medical records and clinical photographs of the cases were obtained from the hospital medical database. The patients' demographics, including age, gender, medical illnesses, past surgical history, family history of HME, main presenting complaint, duration of symptoms, location of the tumor, laterality, physical exam findings, X-ray findings, management, complications, length of follow-up, and recurrence were reviewed. In addition, we have collected the preoperative and postoperative radiological data of the forearm and wrist. First, the ulnar variance (the distance between two horizontal lines drawn across the linear axes of the radius and the ulna from the internal distal physeal plate of the radius and the base of the head of the ulna, respectively, is defined as the distance between two horizontal lines drawn across the linear axes of the radius and the ulna. The normal range is between -2 and +2 millimeters) [15]. Second, the radial articular angle (angle formed by a line drawn parallel to the radius's linear axis and a line drawn parallel to the radius's distal articular surface; a line drawn from the center of the head of the radius to the radial margin of the distal physeal plate defines the radius's linear axis. The normal angle range is between 15 and 30 degrees) [5,6,8,16]. Lastly, the carpal slip (the percentage of lunate bone contact with the radius was used to assess the condition; the lunate is crossed by a line drawn from the center of the olecranon to the ulnar border of the radial epiphysis and a normal carpal slip is defined as an ulnar displacement of the lunate bone of more than 50%) [5,6,8,16]. Figure 1 shows an illustration of the landmarks used to measure the different radiological data included.

The Masada deformity scale was used to classify each patient's forearm [5]. This scale represents both the functional disability as well as the severity of the forearm deformity. The classification divides HME into three types, where type two is divided into two subgroups, A and B. Type I indicates radial head not dislocated, primary exostosis from the distal region of the ulna, ulna relatively short, radius bending. Type IIA shows ulnar shortening, radial head dislocation, and proximal sessile radial exostosis. Type IIB shows radial head ulnar shortening, subluxation, or dislocation without proximal sessile radial exostosis. Lastly, in Type III, the distal section of the radius has a significant exostosis, and the radius has a relative shortening. IBM SPSS Statistics for Windows, Version 24.0 (Released 2016; IBM Corp., Armonk, New York, United States) was used to analyze the data. The respondents were described using descriptive statistics, counts, proportions (percentages), and mean.

## Results

Participants

We identified a total of 10 patients with confirmed HME in the forearm (12 forearms). The average age of the first presentation to our institute was 10.5 ± 5.83 years (range of 2-21 years). The average age of those undergoing surgery was 12.7 ± 5.13 years (a range between 5 and 22 years). Five patients were females, and five were males. Most of the patients had no other medical illnesses, except one who had renal vesicoureteral reflex. Family history was positive among six patients (60%). The average length of follow-up was 62.25 months (a range of 3-121 months). Demographic details are provided in Table 1.

**Table 1:** Demographics and length of follow-up of the included patients

Case	Age (Age at surgery) (years)	Gender	Family history	Length of follow-up (months)
1	2 (11)	Male	Positive	60
2	5 (5)	Female	Negative	121
3	6 (6)	Female	Negative	NA
4	6 (9)	Male	Negative	61
5	10 (10)	Female	Positive	84
6	10 (15)	Male	Positive	25
7	11 (15)	Male	Positive	84
8	16 (16)	Male	Negative	60
9	18 (18)	Female	Positive	3
10	21 (22)	Female	Positive	NA

HME-related variables

The main presenting complaint among the patients was painless deformity (n = 10, 100%). The average duration of symptoms was 6.57 ± 4.27 years (a range of 3-15 years). Among the patients, the most common site of HME was mainly the radius (n = 5, 50%), followed by the ulna, which was observed in twp patients (20%). Regarding the laterality, the left forearm was involved more (n = 6, 60%) than the right (n = 3, 30%). One patient had bilateral HME. Table 2 demonstrates the remaining HME-related clinical data.

**Table 2:** Main presenting complaints, location of tumor, management, and outcome of the included patients LROM: limited range of motion; NA: not available

Case	Primary Complaint at presentation	Duration of symptom (years)	Location of tumor	Masada scale	Laterality	Examination findings	Management given	Outcome
1	Deformity with LROM	3	Sessile radial exostosis	III	Right	Limited supination and pronation.	Excision of exostosis	Good with no complication or recurrence
2	Deformity	NA	Ulnar styloid exostosis and deformity of distal radius	IIA	Left	Limited supination and ulnar deviation of wrist. Radial head dislocated.	Excision of exostosis and corrective osteotomy of radius	Recurrent radial bone deformity and dislocation of the radial head
3	Mass	4	Exostosis between posterior chondroma of distal radius	III	Left	Mass volar distal forearm. Limited pronation.	Excision of exostosis	None
4	Deformity with weakness	NA	Diaphyseal achalasia with radial articular angulation 15 degrees and carpal slip less than 30%.	I	Bilateral (operated on the left)	Elbow flexion contracture 10 degree. Supination to 10 degrees, pronation 50 degrees, actively. Supination 20 degrees, pronation 70 degrees, passively.	Excision of exostosis, application of iliac crest bone graft and K-wire fixation	Osteolysis of bone graft and recurrent of the deformity
5	Deformity with LROM	5	Right: distal exostosis of radius and ulna.	I	Bilateral	Right: Shortening and deformity of forearm.	Excision of exostosis	Good with no complication or recurrence
6	Deformity	5	Exostosis left distal ulna measuring 2.5*2 cm. Subluxation of distal radius.	IIB	Left	10-degree pronation and supination	Radial head resection	Good with no complication or recurrence
7	Deformity with LROM	3	Radial head dislocation with bowing and sital exostosis along with ulnar shortening.	I	Right	NA	Radial head resection	Good with no complication or recurrence
8	Deformity with LROM	15	Left Sessile Radial Exostosis	IIA	Left	Bony prominence of the radial head with shortening of forearm and limited supination.	Radial head resection	Good with no complication or recurrence
9	Deformity	11	Dislocated radial head and exostosis of the middle forearm	I	Left	Swelling middle forearm and dislocated radial head	Radial head resection	Good with no complication or recurrence
10	Deformity	NA	Exostosis of both radius and ulna in addition to dislocated head, short ulna and ulnar deviated wrist.	I	Left	Bowing.	Radial head resection	Good with no complication or recurrence

Regarding the Masada scale, the majority of patients (n = 5, 50%) had Masada type I, two patients had Masada type IIA, one patient had Masada type IIB, and two patients had Masada type III.

Management and outcome

**Figure 2:**
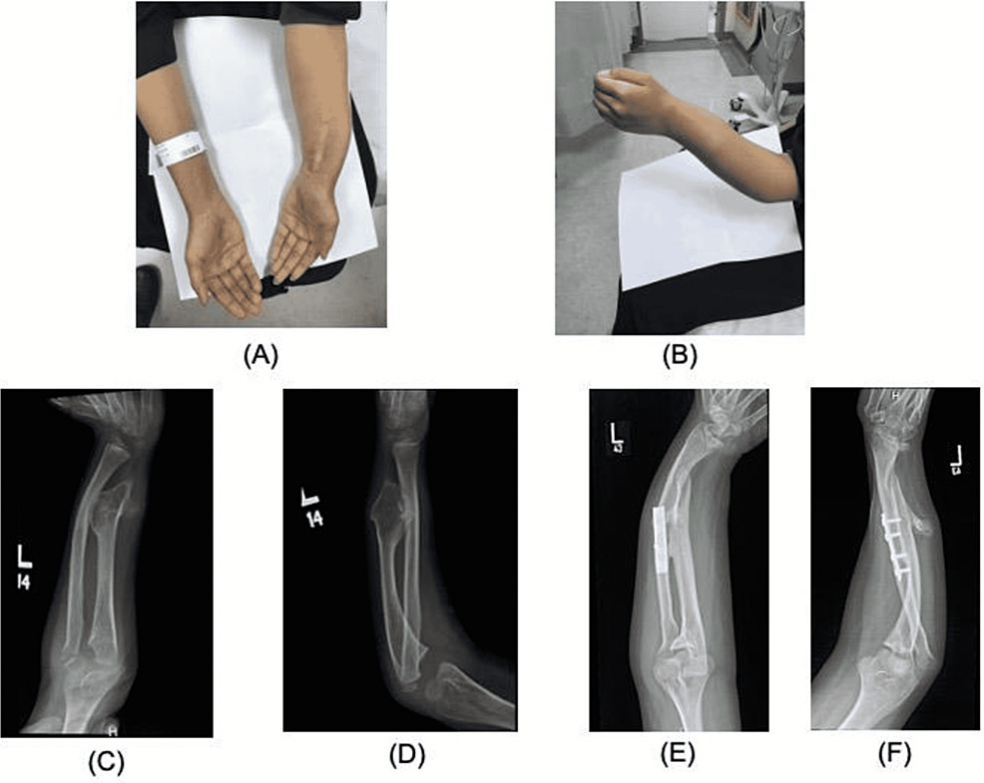
Preoperative and postoperative clinical and radiological images of Case 2 (HME and a forearm deformity, Masada type IIA): (A) Postoperative images of the left forearm show the recurrent radial bone deformity and radial shortening with bowing after six years of the surgery; (B) Another clinical image shows the swelling; (C) Preoperative radiological image showing ulnar styloid exostosis and deformity of the distal radius; (D) Preoperative lateral image; (E) Radiological images following excision of exostosis and corrective osteotomy of the radius six years after the surgery; (F) Lateral view postoperatively HME: hereditary multiple exostosis

Regarding the management, five (50%) underwent radial head resection, three (30%) underwent simple excision of the exostosis by an open method, one (10%) patient underwent excision of the exostosis and corrective osteotomy of the radius, and one (10%) patient underwent excision of the exostosis with the application of iliac crest bone graft and Kirschner wire (K-wire) fixation. The majority of the patients (n = 8, 80%) had no complications or recurrence, except for two patients. The patient (Case 2) who had an excision and corrective osteotomy of the radius developed recurrent radial bone deformity and dislocation of the radial head (Figure 2).

**Figure 3:**
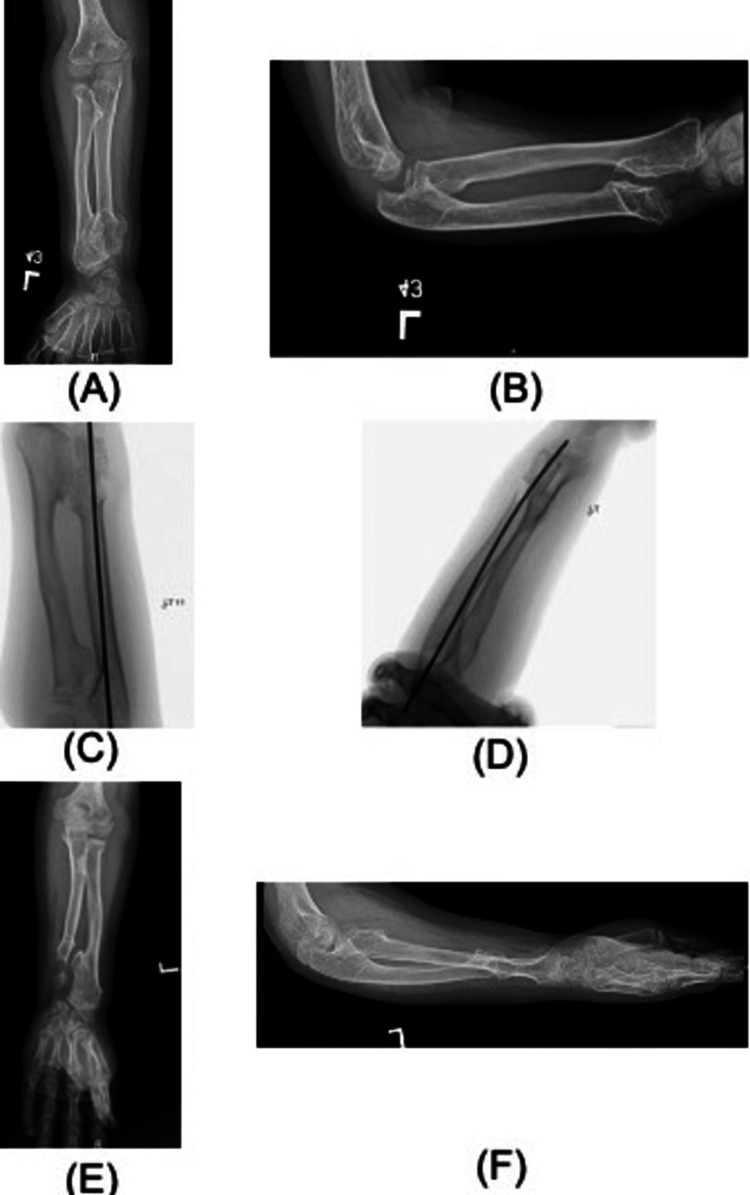
Preoperative, intraoperative, and postoperative radiological images of Case 4 (HME and a forearm deformity - Masada type I): (A) Preoperative image of the left forearm show exostosis with radial articular angulation of 47 degrees and carpal slip of 50%; (B) Preoperative lateral view; (C) Intraoperative images after excision of the exostosis, iliac crest bone graft application, and K-wire fixation; (C) Intraoperative image, including the hand; (D) Intraoperative image lateral view; (E) Radiological image showing almost complete osteolysis of the bone graft and recurrence of the deformity after five years of the surgery; (F) Lateral view postoperative after osteolysis K-wire: Kirschner wire; HME: hereditary multiple exostosis

The other patient who underwent excision with an iliac crest bone graft application developed osteolysis of the bone graft with recurrent deformity (figure 3).

None of the four patients with radial head resection had a posterior interosseous syndrome. For the radiological data, the mean ulnar variance preoperatively was 16.01 degrees, and postoperatively it was 14.58 degrees. On the other hand, the radial articular angle preoperatively was 47.04 degrees, and postoperatively it was 32.22 degrees. The rest of the radiological data is explained in detail in Table 3. 

**Table 3:** Radiographic variables of the included patients, preoperative and postoperative

Case	Ulnar variance - Preoperative (mm)	Ulnar variance - Postoperative (mm)	Radial articular angle – Preoperative (degrees)	Radial articular angle – Postoperative (degrees)	Carpal slip - Preoperative	Carpal slip - Postoperative
1	20	16	55	30	50%	50%
2	23.3	10	55	36	<50%	<50%
3	0	0	20	20	50%	50%
4	15.6	40	47	51.2	50%	<50%
5	16.1	16.6	51	26	> 50%	<50%
6	19.1	11.2	53.2	21	<50%	50%
7	11.1	18	25	36	50%	50%
8	11	10	47	20	50%	50%
9	17.9	5	49	34	<50%	50%
10	26	19	68.2	48	50%	50%

## Discussion

HMEs are a childhood-onset autosomal dominant condition affecting the skeleton. The incidence is estimated to be 1/50,000 at birth, with de novo mutations occurring in 20-30% of cases [1,2]. Bony exostoses are a kind of benign osteocartilaginous growth that begins at the growth plate and causes metaphyseal remodeling, bony abnormalities, and asymmetric long bone development. Thirty to sixty percent of patients have forearm abnormalities, but there is no agreement in the literature on how to treat them and what surgical alternatives are available [3-6]. When the radial head is displaced, common clinical abnormalities include a shorter forearm bending, an ulnar deviation, and an outward protrusion of the radial head. In this paper, we report our experience with the diagnosis, treatment, and natural history of HME of the forearm in ten patients. This study is the first in our region to describe the clinical features and the management of forearm HME.

Regarding the appropriate age to operate on a deformed forearm, there are two conflicting viewpoints in the literature. Several authors advocate early and vigorous surgery, although their patients' average follow-up was just 3.5 years, which seems insufficient to evaluate recurrences [5,6,17-19]. Akita et al. represent the second point of view, which advocates a less aggressive strategy incorporating subsequent surgical operations towards the conclusion of the growth spurt. Their study's longer-term follow-up (13 years) demonstrated persistent recurrence among children who had surgery too early [8]. On the other hand, in our study, the average age of those undergoing surgery was 12.7 years (a range of 5-22 years), with an average length of follow-up of 62.25 months (a range of 3-121 months). In patients with HME forearm involvement, the objective of surgery is to correct the radial deformity and restore radioulnar variance. Recurrence seems inevitable if children are operated on too early since HME is a disease of the growth spurt that continues to progress until skeletal maturity [12]. This can be explained by the fact that early and complete excision of the growth plate's exostosis can interfere with forearm development.

Contrarily, if the exostosis lesion were partially excised in the trial of reserving the growth plate, this would lead to the recurrence of the lesion. In a series of 10 patients, Fogel et al. observed that excision of osteochondromas was ineffective in controlling the deformity progression [6]. However, Masada et al. demonstrated excellent functional results with simple excision in two patients. The procedure was limited to patients with relative radial shortening secondary to distal osteochondromas of the radius [5]. Wood et al. established improved cosmesis after simple excision in four patients, but only minimal improvement in rotation of the forearm [20]. In a study conducted by Litzelmann et al., they found that most radiologic parameters improved significantly postoperatively. However, the recurrence rate was 60% of the forearms during follow-up [12]. The radiological parameter improvements were consistent with our findings, as the mean ulnar variance preoperatively was 16.01, and postoperatively it was 14.58. On the other hand, the radial articular angle preoperatively was 47.04, and postoperatively it was 32.22.

Among the included patients, five (50%) underwent radial head resection. Radial head resection seems to be the most practical way of managing forearm HME. Patients were most satisfied with the results due to removing the main complaint (the forearm swelling caused by the dislocated head), which can be recommended after skeletal maturity to avoid migration. Most of the included patients had no complications or recurrence, except for two patients. The patient who had an excision and corrective osteotomy of the radius developed recurrent radial bone deformity and radial head dislocation. The authors believe that the recurrence in case two was due to excising part of the tumor rather than entirely removing it. The other patient who underwent excision with an iliac crest bone graft application developed osteolysis of the bone graft with recurrent deformity. The decision to proceed with the iliac crest allograft was due to the gap of about 2.5 cm, which was filled by the allograft and stabilized by K-wire for incorporation. Although with this complication, in the last visit, the patient was asymptomatic with no complaints. We believe that lengthening the ulna from the middle third and applying the iliac crest allograft between two normal bones would have been a more favorable decision to prevent the osteolysis in case four.

Even though the study aims have been accomplished and it is considered the first to be conducted in the Middle East, we had several limitations that need to be highlighted. The first limitation is the retrospective nature of this research and the fact that improper documentation was noticed, whether it was a preoperative or postoperative assessment. The second limitation is the small number of patients. This could be explained by HME being a rare condition, as most prior studies involved few patients. The present study's findings suggest that specific indications may need to be revisited, but further research is still required to compare surgically treated individuals to non-operated patients. Another limitation is the short average duration of follow-up as the younger-aged patients require a longer time period of study. Many patients with HME have forearm involvement, but it is not diagnosed until visual deformity has occurred. Thus, most of the patients in this study had radiographs only after their first decade of life. We recommend that future studies include a comparative table including a comprehensive review of the literature regarding the country of the study, the total number of cases, mean duration of follow-up, management strategy, and chances of recurrence.

## Conclusions

We presented an overview of our experience and strategy in treating forearm abnormalities in HME. We have discussed our current surgical correction approach for HME forearm abnormalities. We believe that simple excision of the lesion at skeletal immaturity and follow-up clinically and radiologically is the most practical initial management. If a secondary procedure is needed in the future, simple excision of the dislocated radial head would be the most beneficial for the patient. While the first findings are promising, additional studies are required to identify predictive factors that may influence the outcome and patient satisfaction in surgery for forearm deformities caused by numerous exostoses.
